# Author’s reply (in reference to letter to editor proposed by Etem Caliskan, Catherine J. Pachuk, Louis P. Perrault, Maximilian Y Emmert and entitled: preservation solutions to improve graft patency: *The devil is in the detail*)

**DOI:** 10.1186/s13019-021-01391-4

**Published:** 2021-01-21

**Authors:** Olivier Fouquet, Jean-David Blossier, Simon Dang Van, Pauline Robert, Agnès Barbelivien, Frédéric Pinaud, Patrice Binuani, Maroua Eid, Daniel Henrion, Laurent Loufrani, Christophe Baufreton

**Affiliations:** 1 Department of Cardiac Surgery, CHU, Angers, France; 2 Institute MITOVASC CNRS UMR 6015, INSERM, 1083 Angers, France; 3 Department of Cardiac Surgery, CHU Dupuytren, Limoges, France; 4 University Hospital of Angers, Angers, France

**Keywords:** Venous graft, Graft patency, Preservation solutions

## Abstract

Not applicable.

We appreciate the interest and comments from Dr. Caliskan and colleagues [1] about our recently published article [2]. We fully agree that many confounding factors of our experimental study may alter potential beneficial effects of storage solutions reason that the results must be interpreted with caution. The main criticism formulated by Dr. Caliskan concerns the composition of our referent solution, GALA (Glutathione, L-ascorbic acid, L-arginine and glucose; pH= 7.4). From June 2005, the department of cardiac surgery of the University Hospital of Angers uses the GALA solution in clinical practice. Historically, collaboration has been established with the cardiac surgery and perfusionnist team of Veterans Administration Medical Center of West Roxbury (Harvard Medical School) since 1999. In 2003, Thatte H. et al. showed that duration of storage time in GALA solution (from 1 h to 24 h) did not alter smooth muscle or endothelial cell function [3]. These results constituted a major turning point in our practices for the conservation of venous grafts. The clinical data recently published [4, 5] with the commercially available DuraGraft (Somahlution Inc., Jupiter, Fla) which is formulated based on the GALA solution comforted us in our practice. We have developed our own GALA solution from the formula proposed by the publication of Thatte H [3]. in accordance with European legislation about the injectable solutions. The department of Hospital Pharmacy elaborates the GALA solution with quality controls step by step in order to obtain the most neutral pH possible. We recognize that there is probably a confusion bias in the composition proposed in our article. In fact, each component is mentioned with a unit formula (“the devil is in the details”). Concept solution with these unit formulas must lead to an acid composition. For 1 liter, our solution is initially composed with the following components: 0.67 ml magnesium sulfate (15% amp. 10 ml), 59.5 mg potassium hydrogenophosphate, 1 ml magnesium chloride (10% amp.10 ml), 1.4 ml calcium chloride (10% amp. 10 ml), 1.55 ml L-ascorbic acid (1 g/5 ml), 2.67 ml potassium chloride (15% amp. 10 ml), 10 ml heparin (25,000 UI/5 ml), 20 ml D-glucose (5% (50 ml)), 25 ml sodium bicarbonate (1.4% amp. 10 ml), 40 ml sodium chloride (20% amp. 20 ml), 90 mg reduced glutathione, 150 mg L-arginine, sterile water (sufficient quantity for 1 l). The pH is between 7 and 7.8, buffering is performed in order to obtain a pH closest to 7.4 with a control on agar. Then, the packaging is presented in the form of sterile 30 ml polyethylene bottles. Only the sodium phosphate proposed by Thatte is not found in our solution because of European legislation on the pharmacopoeia of injectable solutions. It is noteworthy to mention that amounts of the GALA main components, as the rationale for efficacy of this solution, are exactly similar between the US and Europe formulations. The results of patent venous graft must be interpreted with caution due to the small sample size. Concerning the occluded grafts in particular in the GALA group, we observed an important intimal hyperplasia with intraluminal thrombus. Similar results with or without venous graft treatment were observed in arteriovenous fistula in murine model at 28 days [6] and at 6 weeks important smooth muscle cells and collagen deposits and macrophage infiltration [7].

In summary, we agree with Caliskan that our experimental study has several limitations and the results have to be taken with caution if we extrapolated to clinical practice. However, the composition of GALA solution elaborated for this experimental study is exactly the same for conservation of venous graft in coronary bypass grafting. Venous graft failure involves multiple factors and long-term follow-up in larger randomized studies is needed to evaluate the effect of storage solutions on clinical outcomes.

## Data Availability

Not applicable.
